# Lead Astray? The Hidden Contaminants in Australian Anabolic–Androgenic Steroid Market and Their Potential Health Impact

**DOI:** 10.1111/dar.70007

**Published:** 2025-07-21

**Authors:** Alison Craven, Jason Ferris, Suzanne Nielsen, Timothy Piatkowski

**Affiliations:** ^1^ PEDTest Australia Perth Australia; ^2^ Centre for Health Services Research, The University of Queensland Brisbane Australia; ^3^ Monash Addiction Research Centre, Eastern Health Clinical School, Monash University Melbourne Australia; ^4^ School of Applied Psychology and Griffith Centre for Mental Health, Griffith University Australia; ^5^ Queensland Injectors Voice for Advocacy and Action Sunshine Coast Australia

**Keywords:** anabolic–androgenic steroids, arsenic, lead, poison, toxic supply

## Abstract

**Introduction:**

Unregulated anabolic–androgenic steroid (AAS) markets are a global phenomenon with significant variability in product composition and purity. This study aimed to determine the chemical composition of AAS sold in Australia.

**Methods:**

This study analysed anonymously donated AAS samples. Samples were chemically analysed by an independent forensic laboratory using gas chromatography–mass spectrometry, liquid chromatography–mass spectrometry and inductively coupled plasma analysis to identify active ingredients, contaminants and heavy metals, with results interpreted in the context of harm reduction.

**Results:**

Analysis of 28 AAS samples (16 injectable, 10 oral, 2 raw powders) revealed that 15 samples were mislabelled or mis‐sold. When considering the 21 samples with clearly defined expected labelled dosages, 4 of these were within a ±5% deviation of the expected purity. Twelve heavy metals were detected in injectable and oral products, with mean concentrations ranging from 0.07 to 62.54 μg/mL in injectables and 1.94–47,901 μg/g in orals. Seven metals were identified in raw powders (mean 23.0 μg/g, range 0.96–51.73 μg/g).

**Discussion and Conclusions:**

The variability and contamination of AAS products pose significant health risks. Implementing a comprehensive surveillance and testing system is essential. This approach would track trends, identify contaminants and provide consumers with real‐time, accurate information to support informed decision‐making and harm reduction.

## 1 Introduction

Unregulated markets for anabolic–androgenic steroids (AAS) are a global phenomenon [1]. However, we have limited understanding of the full range of products circulating within these markets [2, 3]. The presence and purity of AAS are known to vary significantly [2, 4], yet the extent of this variation and the potential contaminants present in these products remain largely unexplored. AAS, commonly used for enhancing appearance, performance and wellbeing [5, 6, 7], are often obtained from unregulated sources, leading to substantial variability in product composition. While the active ingredients may align with labelled content, the possibility of adulteration, substitution and contamination with harmful substances remains a pressing concern.

The presence of cutting agents and unreacted byproducts in AAS [3, 4], both commonly recognised adulterants in unregulated and falsified substances [8], poses significant risks to consumer health. However, beyond these expected contaminants, we currently do not know whether AAS products also contain heavy metals, which introduces a far more alarming health concern. In line with international [9] and Australian [10] guidelines, the Therapeutic Goods Authority sets strict limits on heavy metal impurities in medicines to ensure safety. For example, the permissible daily exposure for lead in orally administered products is 5 μg/day, with similar thresholds for other metals like arsenic and cadmium [10]. These limits aim to minimise consumer exposure to trace contaminants, which are unavoidable but must be kept within strict ranges relative to the dose.

With the increasing prevalence of AAS use globally [11, 12]—estimated to have grown from 1.6% to 4% in the last 10 years among women [11]—there is a critical need to better understand the quality and safety of these substances. However, the gap in knowledge surrounding the full spectrum of substances found in unregulated AAS products presents an urgent challenge for harm reduction strategies, with the only documented trial in the world providing evidence for presence and purity but not other contaminants [13]. Without such information, consumers remain vulnerable to adverse health effects, some of which may be long‐term and irreversible.

This study aimed to determine the chemical composition of AAS sold in Australia. Specifically, the analysis of the provided substances sought to determine the presence and purity of active ingredients and evaluate the presence of cutting agents, residues or heavy metals.

## 2 Methods

### 2.1 Study Design

Samples were provided anonymously by members of the public, with no identifying information recorded. An ethics exemption was granted for this research by Griffith University's Research Ethics Committee.

### 2.2 Sample Collection

People who use AAS anonymously submitted 28 product samples to an independent forensic laboratory (Sharp and Howells Pty Ltd.) for chemical analysis. PEDTest Australia, a private harm reduction business, facilitated and funded the testing, informing the laboratory in advance. Donors, actively engaged in AAS use, provided their expectations about the substances submitted. While these samples, reportedly purchased between 2022 and 2024, may not represent all current batches, they offer insight into the range of products available on the Australian market. The submitted samples comprised a diverse range of products: 16 injectable AAS, 10 oral AAS and 2 raw AAS powders used in AAS manufacture.

### 2.3 Sample Analysis

Upon receiving the samples, the laboratory conducted all chemical analyses and reported the results back to PEDTest Australia for further interpretation and dissemination. The independent laboratory conducted a three‐stage chemical analysis of the samples. Injectable oils (1–5 mL) were diluted in a suitable organic solvent (e.g., methanol) for analysis. Oral tablets (approximately 100 mg) were crushed and dissolved in methanol. Raw powders (approximately 100 mg) were similarly dissolved in methanol. All solutions were filtered before analysis.

#### 2.3.1 Analytical Methods

1. Gas chromatography–mass spectrometry: A PerkinElmer Clarus 680 Gas Chromatograph coupled to a PerkinElmer SQ8 Mass Spectrometer was used. The gas chromatography was equipped with a 30 m × 0.25 mm ID × 0.25 μm film thickness capillary column. Helium served as the carrier gas at a flow rate of 1.0 mL/min. The oven temperature was programmed to optimise the separation of analytes. Electron ionisation mode was employed for mass spectrometric detection. This method was used for initial screening to identify unexpected compounds, contaminants or substitutions.
2. Liquid chromatography–mass spectrometry: Active AAS ingredients, unreacted moieties (chemical fragments or residues from incomplete synthesis) and byproducts were analysed using liquid chromatography–mass spectrometry. The specific instrument and parameters were optimised for the detection of target analytes.
3. Inductively coupled plasma optical emission spectrometry: Heavy metal analysis was performed using a PerkinElmer AVIO 200 inductively coupled plasma optical emission spectrometry. Samples were prepared by acid digestion, and the instrument was calibrated using external standards. Validation followed best‐practice guidelines for laboratory‐based analytical methods, ensuring methodological robustness [14].

Quantification was achieved using external calibration methods. Calibration curves were constructed using known concentrations of standards, and sample concentrations were determined by comparing their responses to the calibration curves [13, 15]. The details of these analyses are included in Table 1. This study extends beyond the technical findings reported by the laboratory by providing a critical interpretation of the results in the context of harm reduction and public health. The independent lab's findings were examined to build on the limited body of knowledge about the Australian AAS market and highlight potential health implications.

**TABLE 1:** Summary characteristics.

Category	Expected compound	Presence issues	Labelled dosage	Purity range (mean ± SD, *n*)
Injectable oils (*n* = 16)	Testosterone enanthate (*n* = 5)	Trestolone acetate detected in one sample. Testosterone cypionate and androstenediol diacetate detected in one sample	200–250 mg/mL	209–245 mg/mL (232 ± 20, 3)
	Boldenone undecylenate (*n* = 4)	One boldenone sample swapped for testosterone enanthate and propionate	200 mg/mL	101–206 mg/mL (161 ± 54, 3)
	Testosterone propionate (*n* = 1)	Testosterone enanthate detected	Not provided	88.84 mg/mL
	Trenbolone acetate (*n* = 2)	Testosterone acetate detected in one sample	Not provided	88 mg/mL
	Nandrolone phenylpropionate (*n* = 1)	Unexpected compound (unknown) detected in one sample	100 mg/mL	83 mg/mL
	Methenolone enanthate (*n* = 1)	Testosterone cypionate found instead in one sample	Not provided	75–190 mg/mL (132 ± 81, 2)
Orals (*n* = 10)	Oxandrolone (*n* = 5)	One sample contained methandrostenolone instead of oxandrolone. One sample contained no compound	10–20 mg	2–6.8 mg (4 ± 2, 3)
	Oxymetholone (*n* = 2)	Both samples replaced with mestanolone	50 mg	Undetected
	Stanozolol (*n* = 2)	No compound present	10–25 mg	Undetected
	Methenolone acetate (*n* = 1)	No unexpected compounds detected	Not provided	5.2 mg
Raw powders (*n* = 2)	Testosterone enanthate (*n* = 2)	Testosterone without ester, androstenedione, testosterone valerate, methandriol, drostanolone enanthate and another unidentified steroid compound detected in both sample	100%	87.0%–96.9% (91.9 ± 3.2)

## 3 Results

The analysis of 28 samples revealed that 15 samples were mislabelled or mis‐sold (see Table 1; see Table S1). When considering the 21 samples with clearly defined expected labelled dosages, 4 were within a ±5% deviation of the expected purity as per seminal work in this area [13, 15]. In the analysis of injectable, oral, and raw powder AAS products, 12 heavy metals were quantifiable in both injectable and oral formulations, while 7 metals were detected in raw powders (see Table 2; see also Tables S2 and S3).

**TABLE 2:** Impurities summary.

Category	Calcium	Magnesium	Boron	Copper	Chromium	Iron	Lead	Aluminium	Arsenic	Cadmium	Zinc
Injectable (μg/mL)											
Av (min–max)	17.38 (8.38–37.64)	43.92 (9.75–78.08)	23.33 (7.46–45.64)	1.00 (0.47–1.34)	1.59 (0.84–4.11)	19.14 (8.08–43.38)	1.57 (1.13–2.01)	47.31 (12.98–110.00)	0.09 (0.09–0.09)	0.07 (0.07–0.07)	62.54 (5.69–199.54)
SD	7.95	48.32	11.99	0.39	1.25	14.77	0.62	24.51	0.00	0.00	78.25
PDE	NL	NL	NL	3000	1100	NL	5	NL	15	2	NL
Oral (μg/g)											
Av (min–max)	47,901.00 (70.00–118,000.00)	1048.57 (390.00–1900.00)	151.31 (13.74–580.00)	6.53 (0.55–15.44)	7.37 (1.04–17.37)	229.46 (26.01–775.04)	1.94 (1.15–3.10)	651.34 (83.42–1220.00)	2.35 (0.52–3.42)	< 5	2.42 (5.17–30.47)
SD	53,543.34	582.88	193.28	4.97	5.05	364.31	0.73	411.10	1.59	0.00	8.66
PDE	NL	NL	NL	3000	11,000	NL	5	NL	15	15	NL
Raw (μg/g)											
Av (min–max)	48.04 (36.94–59.13)	< 5	38.80 (18.47–59.13)	0.96 (0.74–1.18)	2.16 (1.66–2.66)	< 5	1.18 (1.18–1.18)	51.73 (29.57–73.89)	0.37 (0.37–0.37)	< 5	< 5
SD	15.69	0.00	28.75	0.31	0.71	0.00	0.00	31.34	0.00	0.00	0.00
PDE	NL	NL	NL	3000	11,000	NL	5	NL	15	15	NL

*Note*: Averages, standard deviation and range provided for detectable values only; μg/mL (micrograms per millilitre) represents the concentration of a substance in a liquid sample.

Abbreviation: NL, not listed in guideline.

For injectable oils (*n* = 16), several compounds deviated from their labelled dosages. The most common were testosterone enanthate (*n* = 5) and boldenone undecylenate (*n* = 4), alongside trenbolone acetate (*n* = 2), methenolone enanthate (*n* = 2) and nandrolone phenylpropionate (*n* = 1). Testosterone enanthate (labelled 250 mg/mL) ranged from 209 to 245 mg/mL (232 ± 20 mg/mL, *n* = 3), but one sample contained trestolone acetate and one contained testosterone cypionate. Boldenone undecylenate (labelled 200 mg/mL) ranged from 101 to 206 mg/mL (161 ± 54 mg/mL, *n* = 3), with one sample misidentified as testosterone enanthate/propionate. Trenbolone acetate was detected at 88 mg/mL in one sample, while another contained testosterone acetate. Methenolone enanthate (*n* = 2) ranged from 75 to 190 mg/mL (132 ± 81 mg/mL), and nandrolone phenylpropionate (labelled 100 mg/mL) measured 83 mg/mL, with an unknown compound detected.

A total of 10 oral AAS samples were analysed. Oxandrolone was the most commonly identified compound (*n* = 5), but one sample contained methandrostenolone instead and another contained no detectable compound. Oxymetholone (*n* = 2) was entirely replaced with mestanolone and stanozolol was also undetected in both samples. Methenolone acetate (*n* = 1) contained no unexpected compounds, with a measured dosage of 5.2 mg. The purity range for oxandrolone varied from 2 to 6.8 mg. In raw powders (*n* = 2), testosterone enanthate exhibited the presence of multiple unexpected compounds, including androstenedione, Methandriol and Drostanolone, with a purity range from 87.0% to 96.9% (91.9% ± 3.2%).

For injectable products, heavy metal analysis identified a number of elements, of which 1 mil was within PDE guidelines [9], see Table 2 for the relevant list and PDEs. For injectable products, heavy metal analysis identified lead at 1.57 μg/mL, which is below the PDE of 5 μg/day for parenteral use. Cadmium was detected at 0.07 μg/mL, below the parenteral PDE of 2 μg/day, and arsenic at 0.09 μg/mL, also within the 15 μg/day parenteral limit. Among other elements, copper (1.00 μg/mL) was well below its PDE of 300 μg/day, chromium (1.59 μg/mL) was under the 1100 μg/day limit and nickel (1.71 μg/mL) was below the 20 μg/day parenteral threshold.

For oral products, heavy metal analysis identified a number of elements, of which 1 g was within PDE guidelines [9], see Table 2 for the relevant list and PDEs. For oral products, heavy metal analysis (reported in μg/g) detected lead at 1.94 μg/g, remaining below the oral PDE of 5 μg/day if daily intake is under ~2.5 g. Arsenic was found at 2.35 μg/g, within the 15 μg/day oral PDE, and nickel at 27.48 μg/g, which could exceed the 200 μg/day oral PDE depending on dose size. Copper (229.46 μg/g) and chromium (7.37 μg/g) were within their respective PDEs of 3000 and 11,000 μg/day.

## 4 Discussion

This study sought to determine the chemical composition of AAS sold in Australia across 28 products. More than half were mislabelled or mis‐sold, with only four meeting the expected purity within ±5%. These findings are consistent with trends seen internationally: Researchers in Brazil found 42% of AAS were counterfeit, including 65.2% of oil‐based formulations [16], while Fabresse et al. reported 80% of AAS seized from French bodybuilders were incorrectly labelled [17]. In Australia, a community‐led testing trial found 13% of AAS contained different substances than expected [13], reinforcing the global consistency of quality issues identified by Magnolini et al. [1]. The current study also revealed heavy metal contamination in all product types. Although metal contamination is well documented in supplements [18, 19], it remains largely overlooked in AAS. These findings point to a largely unregulated and hazardous AAS market, where consumers are routinely exposed to mislabelled, impure and potentially toxic products.

Pharmaceutical‐grade AAS are manufactured under strict quality control standards, minimising the risk of contamination with heavy metals and other impurities [20]. The detection of heavy metals such as lead, cadmium and arsenic in AAS products, outside of permissible daily exposure limits, represents a critical health risk. Increased exposure to lead can result in severe health issues, including memory impairment, reduced cognitive function and may also contribute to the development of anaemia and cardiovascular disease [21, 22]. Exposure to cadmium is linked to renal damage, hypertension and liver damage [23, 24]. Arsenic is associated with an increased risk of cancer [25]. Although the concentrations of lead and cadmium in this study were relatively low, the cumulative effects of such metals can be harmful over time. Many AAS consumers inject multiple times weekly, while oral compounds are typically taken daily. This extended use significantly heightens the risk of chronic health issues.

The substantial variability in the composition and purity of AAS products is a key concern, but one which has been acknowledged by AAS‐using communities within the Australian context [3, 4]. Consumers relying on labels for dosing may be exposed to unknown compounds, increasing the risk of unanticipated side effects. We note that for oral AAS products, the issue of substitution was even more pronounced than that of injectable compounds, fitting with extant work [4]. This substitution can have significant health consequences, as consumers may unknowingly ingest compounds with different pharmacological properties or side‐effect profiles. Some oral AAS, like oxymetholone and methandrostenolone, are more hepatotoxic due to their alkylated structure [26, 27], which strains the liver, while others, such as oxandrolone, are less harmful due to being excreted both hepatically and renally [28]. Variability in product content increases the risk of harm, including liver and kidney damage, with prolonged use. Some products contain little or no active ingredient (e.g., stanozolol), making consistent dosing difficult. This undermines harm reduction, as consumers may increase their dose to achieve effects, raising the risk of toxicity. Without reliable information, consumers face heightened long‐term health risks.

While this study offers valuable insights into the variability and contamination of illicit AAS, there are several limitations that must be acknowledged. The small sample size, while significant, may not be representative of the broader Australian AAS market. Nonetheless, it is likely that untested AAS substances from an unregulated market are similarly harmful. In addition, the study only focused on products collected from 2022 to 2024. The composition of falsified medicines, which includes AAS, is continuously evolving in response to market dynamics, regulatory changes or shifts in demand or public awareness. Further research should aim to include a larger and more diverse range of sources to paint a more comprehensive picture of the risks associated with unregulated AAS use. While the primary health risks of anabolic steroid use are well‐established and include hormonal, cardiovascular and psychiatric effects [29], this study focuses specifically on the underexplored issue of heavy metal contamination. While we acknowledge broader AAS‐related harms, our work uniquely highlights an additional, less‐recognised risk that may inform harm reduction efforts and testing strategies.

To address the evolving challenges in AAS manufacturing, establishing a robust surveillance and testing would ensure that the latest trends in illicit AAS products are continuously monitored, enabling real‐time updates on potential contaminants and hazards. Such a system could support informed decision‐making and strengthen harm reduction efforts.

## Author Contributions

A.C. and T.P. conceptualised the study. A.C. coordinated data collection and chemical analysis. J.F., S.N., and T.P. verified data and performed data analysis. T.P. drafted the manuscript with input from A.C., S.N., and J.F. All authors reviewed, revised, and approved the final manuscript and had final responsibility for the decision to submit for publication.

## Disclosure

Alison Craven is the CEO of PEDTest Australia, a lived‐living experience‐led, privately owned harm reduction organisation which provides testing kits to people in Australia who use anabolic–androgenic steroids and wish to have more information regarding their substance.

## Conflicts of Interest

PEDTest Australia is a private business, led by CEO Alison Craven, that provides reagent testing kits for people who use drugs. PEDTest Australia funded the independent testing of anabolic–androgenic steroid samples submitted by the community, the results of which are presented in this study. Alison Craven is also the author of this work. Suzanne Nielsen is supported by a National Health and Medical Research Centre Investigator Grant (2025894). The other authors declare no conflicts of interest.

## Supporting information


**Table S1.** Presence and purity of compounds submitted or analysis.
**Table S2.** Impurities analysis.
**Table S3.** Impurities table extended.

## Data Availability

The data that supports the findings of this study are available in the Supporting Information of this article.
