# Supplementary material for: Lead Astray? The Hidden Contaminants in Australian Anabolic–Androgenic Steroid Market and Their Potential Health Impact
Source: Drug Alcohol Rev. 2025 Jul 21;44(6):1641–7. doi: 10.1111/dar.70007 (PMC12405805; doi:10.1111/dar.70007)
Supplement: Supplementary file 1 — Table S1. Presence and purity of compounds submitted or analysis. Table S2. Impurities analysis. Table S3. Impurities table extended. [file DAR-44-1641-s001.docx]

**Supporting Information**

**Table S1. Presence and purity of compounds submitted or analysis**

| **Sample No** | **Group** | **Labelled active chemical** | **Labelled dosage** | **Results of analysis** | **Labelled dosage** | **Concentration determined by analysis (mg/ml )** |
| --- | --- | --- | --- | --- | --- | --- |
| 1 | Large Volume Oils | Testosterone Enanthate | 250 mg/ml | Benzyl Alcohol |  | 12 |
|  |  |  |  | Benzyl Benzoate |  | 174 |
|  |  |  |  | Trestolone Acetate |  | 1 |
|  |  |  |  | Testosterone Enanthate | 250 mg/ml | 245 |
|  |  |  |  | plus Fatty Acid Esters |  |  |
|  |  |  |  |  |  |  |
| 2 | Large Volume Oils | Testosterone Enanthate | 250 mg/ml | Benzyl Alcohol |  | 10 |
|  |  |  |  | Benzyl Benzoate |  | 116 |
|  |  |  |  | Trestolone Acetate |  | 2 |
|  |  |  |  | Testosterone Enanthate | 250 mg/ml | 243 |
|  |  |  |  | plus Fatty Acid Esters |  |  |
|  |  |  |  |  |  |  |
| 3 | Large Volume Oils | Testosterone Enanthate | 250 mg/ml | Benzyl Alcohol |  | 4 |
|  |  |  |  | Benzyl Benzote |  | 235 |
|  |  |  |  | Androstenediol diacetate |  | 2 |
|  |  |  |  | Testosterone Cyprionate |  | 210 |
|  |  |  |  | plus Fatty Acid Esters |  |  |
|  |  |  |  | Testosterone Enanthate | 250 mg/ml | Not detected |
|  |  |  |  |  |  |  |
| 4 | Large Volume Oils | Boldenone Undecylenate | 200 mg/ml | Benzyl Alcohol |  | 6 |
|  |  |  |  | Benzyl Benzoate |  | 121 |
|  |  |  |  | Boldenone Undecylenate | 200 mg/ml | 175 |
|  |  |  |  | plus Fatty Acid Esters |  |  |
|  |  |  |  |  |  |  |
| 5 | Large Volume Oils | Boldenone Undecylenate | 200 mg/ml | Benzyl Alcohol |  | 11 |
|  |  |  |  | Benzyl Benzoate |  | 202 |
|  |  |  |  | Boldenone Undecylenate | 200 mg/ml | 206 |
|  |  |  |  | plus Fatty Acid Esters |  |  |
|  |  |  |  |  |  |  |
| 6 | Large Volume Oils | Nandrolone phenylpropionate | 100 mg/ml | Benzyl Alcohol |  | 9 |
|  |  |  |  | Benzyl Benzoate |  | 190 |
|  |  |  |  | Nandrolone phenylpropionate | 100 mg/ml | 83 |
|  |  |  |  | plus Fatty Acid Esters |  |  |
|  |  |  |  |  |  |  |
| 7 | Small Volume Oils | Testosterone Propionate | Not provided | Benzyl Alcohol |  | 9 |
|  |  |  |  | Benzyl Benzoate |  | 174 |
|  |  |  |  | Testosterone Enanthate |  | 89 |
|  |  |  |  | plus Fatty Acid Esters |  |  |
|  |  |  |  | Testosterone Propionate | Not provided | Not detected |
|  |  |  |  |  |  |  |
| 8 | Small Volume Oils | Testosterone Enanthate | 200 mg/ml | Benzyl Alcohol |  | 34 |
|  |  |  |  | Benzyl Benzoate |  | 178 |
|  |  |  |  | Trenbolone Enanthate |  | 159 |
|  |  |  |  | plus Fatty Acid Esters |  |  |
|  |  |  |  | Testosterone Enanthate | 200 mg/ml | Not detected |
|  |  |  |  |  |  |  |
| 9 | Small Volume Oils | Trenbolone Acetate | Not provided | Benzyl Alcohol |  | 18 |
|  |  |  |  | Benzyl Benzoate |  | 263 |
|  |  |  |  | Trenbolone Acetate | Not provided | 88 |
|  |  |  |  | plus Fatty Acid Esters |  |  |
|  |  |  |  |  |  |  |
| 10 | Small Volume Oils | Testosterone Enanthate | 250 mg/ml | Benzyl Alcohol |  | 9 |
|  |  |  |  | Benzyl Benzoate |  | 91 |
|  |  |  |  | Testosterone Enanthate | 250 mg/ml | 209 |
|  |  |  |  | plus Fatty Acid Esters |  |  |
|  |  |  |  |  |  |  |
| 11 | Small Volume Oils | Trenbolone Acetate | Not provided | Benzyl Alcohol |  | 2 |
|  |  |  |  | Benzyl Benzoate |  | 83 |
|  |  |  |  | Testosterone Acetate |  | 5 |
|  |  |  |  | Trenbolone Acetate | Not provided | 2 |
|  |  |  |  | plus Fatty Acid Esters |  |  |
|  |  |  |  |  |  |  |
| 12 | Small Volume Oils | Boldenone Undecylenate | 200 mg/ml | Benzyl Alcohol |  | 6 |
|  |  |  |  | Benzyl Benzoate |  | 132 |
|  |  |  |  | Boldenone Undecylenate | 200 mg/ml | 101 |
|  |  |  |  | plus Fatty Acid Esters |  |  |
|  |  |  |  |  |  |  |
| 13 | Small Volume Oils | Boldenone Undecylenate | 200 mg/ml | Benzyl Alcohol | 200 mg/ml | 4 |
|  |  |  |  | Benzyl Benzoate |  | 85 |
|  |  |  |  | Testosterone Propionate |  | 28 |
|  |  |  |  | Testosterone Enanthate |  | 94 |
|  |  |  |  | plus Fatty Acid Esters |  |  |
|  |  |  |  | Boldenone Undecylenate | 200 mg/ml | Not detected |
|  |  |  |  |  |  |  |
| 14 | Small Volume Oils | Methenolone Enanthate | 100 mg/ml | Benzyl Alcohol |  | 11 |
|  |  |  |  | Benzyl Benzoate |  | 139 |
|  |  |  |  | Testosterone cypionate |  | 101 |
|  |  |  |  | plus Fatty Acid Esters |  |  |
|  |  |  |  | Methenolone Enanthate | 100 mg/ml | Not detected |
|  |  |  |  |  |  |  |
| 15 | Small Volume Oils | Methenolone Enanthate | Not provided | Benzyl Alcohol |  | 12 |
|  |  |  |  | Benzyl Benzoate |  | 167 |
|  |  |  |  | Methenolone Enanthate | Not provided | 75 |
|  |  |  |  | plus Fatty Acid Esters |  |  |
|  |  |  |  |  |  |  |
| 16 | Small Volume Oils | Methenolone Enanthate | Not provided | Benzyl Alcohol |  | 9 |
|  |  |  |  | Benzyl Benzoate |  | 148 |
|  |  |  |  | Methenolone Enanthate | Not provided | 190 |
|  |  |  |  | plus Fatty Acid Esters |  |  |
|  |  |  |  |  |  |  |
| 17 | Orals (Tablet) | Oxandrolone | 20 mg | Methandrostenolone |  | 0.96 |
|  |  |  |  | Oxandrolone | 20 mg | Not detected |
|  |  |  |  |  |  |  |
| 18 | Orals (Capsule) | Stanozolol | 25 mg | No peaks detected |  | No peaks detected |
|  |  |  |  |  |  |  |
| 19 | Orals (Tablet) | Methenolone acetate | Not provided | Methenolne acetate | Not provided | 5.21 |
|  |  |  |  |  |  |  |
| 20 | Orals (Tablet) | Oxymetholone | 50 mg | Mestanolone |  | 1.8 mg |
|  |  |  |  | Oxmetholone | 50 mg | Not detected |
|  |  |  |  |  |  |  |
| 21 | Orals (Tablets ) | Oxymetholone | 50 mg | Mestanolone |  | 0.53 |
|  |  |  |  | Oxymetholone | 50 mg | Not detected |
|  |  |  |  |  |  |  |
| 22 | Orals (Tablets) | Oxandrolone | 20 mg | Oxandrolone | 20 mg | 4.06 |
|  |  |  |  |  |  |  |
| 23 | Orals (Tablets) | Oxandrolone | Not provided | No peaks detected |  | Not detected |
|  |  |  |  |  |  |  |
| 24 | Orals (Capsule) | Oxandrolone | 10 mg | oxandrolone | 10 mg | 6.80 |
|  |  |  |  |  |  |  |
| 25 | Orals (Capsule) | Oxandrolone | Not provided | oxandrolone | Not provided | 2.00 |
|  |  |  |  |  |  |  |
| 26 | Orals (Tablet) | Stanozolol | 10 mg | No peaks detected |  | No peaks detected |
|  |  |  |  |  |  |  |
| 27 | RAW Sample A | Testosterone Enanthate | 100% | Testosterone |  | 0.50% |
|  |  |  |  | Androstenedione |  | 0.02% |
|  |  |  |  | Testosterone Valerate |  | 0.70% |
|  |  |  |  | Methandriol |  | 0.50% |
|  |  |  |  | Epitestosterone Butyrate |  | 0.20% |
|  |  |  |  | Testosterone Enanthate | 100% | 87.0% |
|  |  |  |  | Drostanolone Enanthate |  | 9.4% |
|  |  |  |  | Unknown? Similar to Drostanolone Enanthate |  | 0.8% |
|  |  |  |  |  |  |  |
| 28 | RAW Sample B | Testosterone Enanthate | 100% | Testosterone Enanthate | 100% | 96.9% |
|  |  |  |  | Testosterone Valerate |  | 0.9% |
|  |  |  |  | Drostanolone Enanthate |  | 1.2% |

Table S2. Impurities Analysis

| **Sample No** | **Active chemical** | **Calcium** | **Magnesium** | **Boron** | **Cobalt** | **Barium** | **Lithium** | **Beryllium** | **Manganese** | **Copper** | **Chromium** |
| --- | --- | --- | --- | --- | --- | --- | --- | --- | --- | --- | --- |
| 1 | Testosterone Enanthate | 23 | <5 | 15 | <5 | <5 | <5 | <5 | <5 | 1.2 | 1.2 |
| 2 | Testosterone Enanthate | <5 | <5 | 32 | <5 | <5 | <5 | <5 | <5 | <0.5 | <0.5 |
| 3 | Testosterone Enanthate | 16 | <5 | 24 | <5 | <5 | <5 | <5 | <5 | <0.5 | <0.5 |
| 4 | Boldenone undecylenate | 15 | <5 | 7 | <5 | <5 | <5 | <5 | <5 | <0.5 | <0.5 |
| 5 | Boldenone undecylenate | 26 | 78 | <5 | <5 | <5 | <5 | <5 | <5 | 1.0 | 1.0 |
| 6 | Nandrolone Phenylpropionate | 18 | 10 | <5 | <5 | <5 | <5 | <5 | <5 | <0.5 | 1.0 |
| 7 | Testosterone Propionate | <5 | <5 | <5 | <5 | <5 | <5 | <5 | <5 | <0.5 | <0.5 |
| 8 | Testosterone Enanthate | 12 | <5 | <5 | <5 | <5 | <5 | <5 | <5 | <0.5 | <0.5 |
| 9 | Trenbolone Acetate | 13 | <5 | <5 | <5 | <5 | <5 | <5 | <5 | <0.5 | <0.5 |
| 10 | Testosterone Enanthate | 17 | <5 | <5 | <5 | <5 | <5 | <5 | <5 | 1.3 | 1.3 |
| 11 | Trenbolone Acetate | 23 | <5 | 46 | <5 | <5 | <5 | <5 | <5 | <0.5 | 4.1 |
| 12 | Boldenone undecylenate | 17 | <5 | 17 | <5 | <5 | <5 | <5 | <5 | <0.5 | <0.5 |
| 13 | Boldenone undecylenate | 9 | <5 | 28 | <5 | <5 | <5 | <5 | <5 | 0.5 | 0.8 |
| 14 | Methenolone Enanthate | 9 | <5 | <5 | <5 | <5 | <5 | <5 | <5 | <0.5 | <0.5 |
| 15 | Methenolone Enanthate | 38 | <5 | <5 | <5 | <5 | <5 | <5 | <5 | <0.5 | <0.5 |
| 16 | Methenolone Enanthate | 8 | <5 | 17 | <5 | <5 | <5 | <5 | <5 | <0.5 | <0.5 |
| 17 | Oxandrolone | 118000 | 1060 | 76 | <5 | <5 | <5 | <5 | <5 | 1.9 | 3.4 |
| 18 | Stanozolol | 390 | <5 | 580 | <5 | <5 | <5 | <5 | <5 | 15.4 | 17.4 |
| 19 | Methenolone Acetate | 103000 | 1090 | 57 | <5 | <5 | <5 | <5 | <5 | 4.6 | 4.6 |
| 20 | Oxymetholone | 103000 | 1900 | 145 | <5 | <5 | <5 | <5 | <5 | 11.6 | 13.1 |
| 21 | Oxymetholone | 108000 | 760 | 14 | <5 | <5 | <5 | <5 | <5 | 0.5 | 1.9 |
| 22 | Oxandrolone | 2200 | 1700 | 83 | <5 | <5 | <5 | <5 | <5 | 5.8 | 7.5 |
| 23 | Oxandrolone | 160 | 390 | <5 | <5 | <5 | <5 | <5 | <5 | 10.1 | 7.8 |
| 24 | Oxandrolone | 190 | <5 | 104 | <5 | <5 | <5 | <5 | <5 | 9.4 | 9.4 |
| 25 | Oxandrolone | 70 | <5 | <5 | <5 | <5 | <5 | <5 | <5 | 5.1 | 7.7 |
| 26 | Stanozolol | 44000 | 440 | <5 | <5 | <5 | <5 | <5 | <5 | 0.8 | 1.0 |
| 27 | Testosterone Enanthate | 37 | <5 | 18 | <5 | <5 | <5 | <5 | <5 | 0.7 | 1.7 |
| 28 | Testosterone Enanthate | 59 | <5 | 59 | <5 | <5 | <5 | <5 | <5 | 1.2 | 2.7 |

Table S2 Cont.

| **PEDTEST Sample No** | **Active chemical** | **Iron** | **Lead** | **Aluminium** | **Arsenic** | **Cadmium** | **Strontium** | **Antimony** | **Nickel** | **Mercury** | **Zinc** |
| --- | --- | --- | --- | --- | --- | --- | --- | --- | --- | --- | --- |
| 1 | Testosterone Enanthate | <5 | <0.5 | 23 | <0.05 | <0.05 | <5 | <5 | 2.3 | <0.05 | <5 |
| 2 | Testosterone Enanthate | <5 | <0.5 | 13 | <0.05 | <0.05 | <5 | <5 | <0.5 | <0.05 | <5 |
| 3 | Testosterone Enanthate | 8 | <0.5 | 32 | <0.05 | <0.05 | <5 | <5 | <0.5 | <0.05 | 28 |
| 4 | Boldenone undecylenate | <5 | <0.5 | 52 | <0.05 | <0.05 | <5 | <5 | <0.5 | <0.05 | <5 |
| 5 | Boldenone undecylenate | 43 | 1.1 | 61 | <0.05 | <0.05 | <5 | <5 | <0.5 | <0.05 | 200 |
| 6 | Nandrolone Phenylpropionate | 9 | <0.5 | 35 | <0.05 | <0.05 | <5 | <5 | <0.5 | <0.05 | 28 |
| 7 | Testosterone Propionate | <5 | <0.5 | 39 | <0.05 | <0.05 | <5 | <5 | <0.5 | <0.05 | <5 |
| 8 | Testosterone Enanthate | <5 | <0.5 | 35 | <0.05 | <0.05 | <5 | <5 | <0.5 | <0.05 | <5 |
| 9 | Trenbolone Acetate | <5 | <0.5 | 66 | <0.05 | <0.05 | <5 | <5 | 1.3 | <0.05 | <5 |
| 10 | Testosterone Enanthate | <5 | 2.0 | 50 | <0.05 | 0.07 | <5 | <5 | <0.5 | <0.05 | 5.7 |
| 11 | Trenbolone Acetate | 23 | <0.5 | 110 | <0.05 | <0.05 | <5 | <5 | <0.5 | <0.05 | <5 |
| 12 | Boldenone undecylenate | <5 | <0.5 | 83 | <0.05 | <0.05 | <5 | <5 | <0.5 | <0.05 | <5 |
| 13 | Boldenone undecylenate | <5 | <0.5 | 47 | 0.09 | <0.05 | <5 | <5 | <0.5 | <0.05 | <5 |
| 14 | Methenolone Enanthate | <5 | <0.5 | 18 | <0.05 | <0.05 | <5 | <5 | <0.5 | <0.05 | 51 |
| 15 | Methenolone Enanthate | 13 | <0.5 | 50 | <0.05 | <0.05 | <5 | <5 | 1.5 | <0.05 | <5 |
| 16 | Methenolone Enanthate | <5 | <0.5 | 42 | <0.05 | <0.05 | <5 | <5 | <0.5 | <0.05 | <5 |
| 17 | Oxandrolone | 76 | <0.5 | 760 | <0.05 | <0.05 | <5 | <5 | <0.5 | <0.05 | <5 |
| 18 | Stanozolol | <5 | <0.5 | 1200 | <0.05 | <0.05 | <5 | <5 | 1.9 | <0.5 | 14 |
| 19 | Methenolone Acetate | <5 | 1.1 | 290 | <0.05 | <0.05 | <5 | <5 | 1.7 | <0.5 | 5.2 |
| 20 | Oxymetholone | <5 | <0.5 | 730 | <0.05 | <0.05 | <5 | <5 | 1.5 | <0.5 | 30 |
| 21 | Oxymetholone | 41 | <0.5 | 1220 | <0.05 | <0.05 | 27 | <5 | <0.5 | <0.5 | <5 |
| 22 | Oxandrolone | <5 | 1.7 | 83 | <0.05 | <0.05 | <5 | <5 | 1.7 | <0.5 | 7.5 |
| 23 | Oxandrolone | 775 | 3.1 | 1090 | <0.05 | <0.05 | <5 | <5 | 1.6 | <0.5 | 8.5 |
| 24 | Oxandrolone | <5 | 2.1 | 420 | 3.12 | <0.05 | <5 | <5 | 2.1 | <0.5 | 7.3 |
| 25 | Oxandrolone | <5 | 1.7 | 430 | 3.42 | <0.05 | <5 | <5 | 0.9 | <0.5 | 15 |
| 26 | Stanozolol | 26 | <0.5 | 290 | 0.52 | <0.05 | <5 | <5 | <0.5 | <0.1 | <5 |
| 27 | Testosterone Enanthate | <5 | <0.5 | 74 | 0.37 | <0.05 | <5 | <5 | <0.5 | <0.1 | <5 |
| 28 | Testosterone Enanthate | <5 | 1.2 | 30 | <0.05 | <0.05 | <5 | <5 | <0.5 | <0.1 | <5 |

**Table S3. Impurities Table Extended**

| **Category** |  | **Calcium** | **Magnesium** | **Boron** | **Cobalt** | **Barium** | **Lithium** | **Beryllium** | **Manganese** | **Copper** | **Chromium** | **Iron** | **Lead** | **Aluminium** | **Arsenic** | **Cadmium** | **Strontium** | **Antimony** | **Nickel** | **Mercury** | **Zinc** |
| --- | --- | --- | --- | --- | --- | --- | --- | --- | --- | --- | --- | --- | --- | --- | --- | --- | --- | --- | --- | --- | --- |
| **Injectable (ug/ml)** | **Av** | 17.38 | 43.92 | 23.33 | <5 | <5 | <5 | <5 | <5 | 1.00 | 1.59 | 19.14 | 1.57 | 47.31 | 0.09 | 0.07 | <5 | <5 | 1.71 | <5 | 62.54 |
|  | **SD** | 7.95 | 48.32 | 11.99 | 0.00 | 0.00 | 0.00 | 0.00 | 0.00 | 0.39 | 1.25 | 14.77 | 0.62 | 24.51 | 0.00 | 0.00 | 0.00 | 0.00 | 0.53 | 0.00 | 78.25 |
|  | **Min** | 8.38 | 9.75 | 7.46 | <5 | <5 | <5 | <5 | <5 | 0.47 | 0.84 | 8.08 | 1.13 | 12.98 | 0.09 | 0.07 | <5 | <5 | 1.32 | <5 | 5.69 |
|  | **Max** | 37.64 | 78.08 | 45.64 | <5 | <5 | <5 | <5 | <5 | 1.34 | 4.11 | 43.38 | 2.01 | 110.00 | 0.09 | 0.07 | <5 | <5 | 2.31 | <5 | 199.54 |
|  | **PDE** | NL | NL | NL | 5 | 700 | 250 | NL | NL | 3000 | 1100 | NL | 5 | NL | 15 | 2 | NL | 90 | 20 | 3 | NL |
| **Oral (ug/gm)** | **Av** | 47901.00 | 1048.57 | 151.31 | <5 | <5 | <5 | <5 | <5 | 6.53 | 7.37 | 229.46 | 1.94 | 651.34 | 2.35 | <5 | 27.48 | <5 | 1.61 | <5 | 12.42 |
|  | **SD** | 53543.34 | 582.88 | 193.28 | 0.00 | 0.00 | 0.00 | 0.00 | 0.00 | 4.97 | 5.05 | 364.31 | 0.73 | 411.10 | 1.59 | 0.00 | 0.00 | 0.00 | 0.40 | 0.00 | 8.66 |
|  | **Min** | 70.00 | 390.00 | 13.74 | <5 | <5 | <5 | <5 | <5 | 0.55 | 1.04 | 26.01 | 1.15 | 83.42 | 0.52 | 0.00 | 27.48 | 0.00 | 0.85 | <5 | 5.17 |
|  | **Max** | 118000.00 | 1900.00 | 580.00 | <5 | <5 | <5 | <5 | <5 | 15.44 | 17.37 | 775.04 | 3.10 | 1220.00 | 3.42 | 0.00 | 27.48 | 0.00 | 2.08 | <5 | 30.47 |
|  | **PDE** | NL | NL | NL | 50 | 1400 | 550 | NL | NL | 3000 | 11000 | NL | 5 | NL | 15 | 15 | NL | 1200 | 200 | 30 | NL |
| **Raw (ug/gm)** | **Av** | 48.04 | <5 | 38.80 | <5 | <5 | <5 | <5 | <5 | 0.96 | 2.16 | <5 | 1.18 | 51.73 | 0.37 | <5 | <5 | <5 | <5 | <5 | <5 |
|  | **SD** | 15.69 | 0.00 | 28.75 | 0.00 | 0.00 | 0.00 | 0.00 | 0.00 | 0.31 | 0.71 | 0.00 | 0.00 | 31.34 | 0.00 | 0.00 | 0.00 | 0.00 | 0.00 | 0.00 | 0.00 |
|  | **Min** | 36.94 | <5 | 18.47 | <5 | <5 | <5 | <5 | <5 | 0.74 | 1.66 | <5 | 1.18 | 29.57 | 0.37 | <5 | <5 | <5 | <5 | <5 | 0.00 |
|  | **Max** | 59.13 | <5 | 59.13 | <5 | <5 | <5 | <5 | <5 | 1.18 | 2.66 | <5 | 1.18 | 73.89 | 0.37 | <5 | <5 | <5 | <5 | <5 | 0.00 |

NL, not listed in guideline; PDE, permitted daily exposure.
